# The Human Neutrophil Subsets Defined by the Presence or Absence of OLFM4 Both Transmigrate into Tissue *In Vivo* and Give Rise to Distinct NETs *In Vitro*

**DOI:** 10.1371/journal.pone.0069575

**Published:** 2013-07-29

**Authors:** Amanda Welin, Firoozeh Amirbeagi, Karin Christenson, Lena Björkman, Halla Björnsdottir, Huamei Forsman, Claes Dahlgren, Anna Karlsson, Johan Bylund

**Affiliations:** The Phagocyte Research Group, Department of Rheumatology and Inflammation Research, University of Gothenburg, Gothenburg, Sweden; University of Tübingen, Germany

## Abstract

Neutrophil heterogeneity was described decades ago, but it could not be elucidated at the time whether the existence of different neutrophil subsets had any biological relevance. It has been corroborated in recent years that neutrophil subsets, defined by differential expression of various markers, are indeed present in human blood, calling for renewed attention to this question. The expression of the granule protein olfactomedin 4 (OLFM4) has been suggested to define two such neutrophil subsets. We confirm the simultaneous presence of one OLFM4-positive and one OLFM4-negative neutrophil subpopulation as well as the localization of the protein to specific granules. *In vitro*, these neutrophil subsets displayed equal tendency to undergo apoptosis and phagocytose bacteria. In addition, the subpopulations were recruited equally to inflammatory sites *in vivo*, and this was true both in an experimental model of acute inflammation and in naturally occurring pathological joint inflammation. In line with its subcellular localization, only limited OLFM4 release was seen upon *in vivo* transmigration, and release through conventional degranulation required strong secretagogues. However, extracellular release of OLFM4 could be achieved upon formation of neutrophil extracellular traps (NETs) where it was detected only in a subset of the NETs. Although we were unable to demonstrate any functional differences between the OLFM4-defined subsets, our data show that different neutrophil subsets are present in inflamed tissue *in vivo*. Furthermore, we demonstrate NETs characterized by different markers for the first time, and our results open up for functions of OLFM4 itself in the extracellular space through exposure in NETs.

## Introduction

Neutrophil granulocytes develop from myeloid precursor cells in the bone marrow. The cells are then mobilized and released into the bloodstream, where they circulate until prompted to transmigrate into sites of tissue injury or infection or cleared by age. The mature peripheral blood neutrophils contain a large number of preformed secretory organelles of variable size, protein content, and function. Four different secretory organelle subsets termed azurophil granules, specific granules, gelatinase granules and secretory vesicles are formed and loaded with newly biosynthesized proteins sequentially during myelopoeisis. When the cell transmigrates into tissue and becomes activated, the ease with which these different granule populations are mobilized varies, with degranulation occurring in the opposite order to that in which the granules were formed [1,2].

Neutrophils from healthy individuals have historically been viewed as a homogenous population of cells that carry out the same functions, display the same properties during inflammation and in response to infection, and are equipped with the same granule proteins. However, neutrophil heterogeneity in terms of protein expression was discovered several decades ago, without convincing evidence to claim that the existence of different neutrophil subsets was biologically relevant [3]. More recent findings concerning the presence in circulation of distinct subsets of neutrophils, characterized by the expression of different markers, prompt renewed attention to this question [4,5]. One marker that defines two neutrophil subsets in humans is CD177 [6], and another is olfactomedin 4 (OLFM4), very recently described by Clemmensen et al. [7]. OLFM4 is a matrix glycoprotein expressed in prostate, small intestine, colon, stomach, and bone marrow tissue of humans [8]. All myelopoietic cells in the bone marrow of healthy individuals contain and express the *OLFM4* gene, most strongly at the myelocyte/metamyelocyte stage of differentiation. In accordance with the time for OLFM4 synthesis in the bone marrow, the protein is stored in the specific granules. In blood neutrophils, OLFM4 defines two distinct subpopulations; one containing OLFM4 while the other does not. The OLFM4-positive proportion varies between 10–30% of circulating neutrophils in different individuals, and the bimodal protein expression despite uniform mRNA levels indicates that post-transcriptional regulation is responsible [7]. It is known that OLFM4 is upregulated during, and plays a role in, tumorigenesis [9], and mouse studies have indicated a negative role of OLFM4 during infection [10,11]. However, it is not known whether OLFM4 marks a neutrophil subpopulation with a specific purpose, and the function of the OLFM4 protein itself in human neutrophils has not been studied.

Here, we confirm the existence of two subpopulations, defined by the expression of OLFM4, in human blood, as well as specific granule localization of the protein. The subpopulations did not display any differences in terms of proneness to undergo apoptosis or ability to phagocytose bacteria, nor in their propensity to transmigrate into tissue in response to inflammatory stimuli. Nevertheless, we show that both subpopulations are present in extravascular tissues – sites where neutrophils typically carry out their functions. That OLFM4 was present after *in vivo* transmigration to tissues is in line with the subcellular localization of the protein and strong secretagogic stimulation was required to attain conventional secretion by degranulation. Release of granule proteins can also occur via non-classical secretion e.g., through the formation of neutrophil extracellular traps (NETs) [12]. When neutrophils were induced to form NETs *in vitro*, OLFM4 could be detected extracellularly, associated with DNA, and in contrast to other NET constituents, OLFM4 was present in only a subpopulation of the NETs. Although no direct functional differences between the neutrophil subsets were found, our data show that the two neutrophil subtypes, defined by OLFM4 expression, are present in tissue and that OLFM4 can be released from the cells through NET formation, which opens up for potential extracellular functions/effects by the protein. 

## Materials and Methods

### Ethics statement

The study was approved by the Regional Ethical Board of Gothenburg, Sweden (No. 543-07 and No. S010-03). Written informed consent was obtained from the participants.

### Neutrophil isolation from blood and tissue

Blood neutrophils were separated from heparinized whole blood or one day-old buffy coats from healthy blood donors by dextran sedimentation and Ficoll-paque centrifugation according to Boyum et al. [13,14]. After separation, the neutrophils were washed twice and resuspended in Krebs-Ringer glucose (KRG) phosphate buffer supplemented with Ca^2+^ (1 mM) and stored on ice until use. The typical purity of the polymorphonuclear leukocytes obtained by this method was around 95%. Neutrophils isolated from whole blood were used in experiments that quantified the OLFM4-defined subpopulations, while buffy coat cells (which also displayed bimodal expression of OLFM4) were routinely used for functional assays.

*In vivo*-transmigrated neutrophils were obtained by two different approaches. Firstly, after written informed consent, skin chamber neutrophils were obtained from three healthy volunteers. The skin chamber technique is an experimental model of acute inflammation where blisters are formed on the forearm of the healthy volunteer by negative pressure, the blister roofs are subsequently removed and a collection chambers containing autologous serum are applied to the lesions. Neutrophils are then allowed to transmigrate into the collection chambers over 24 h. Details on skin chamber methodology are given in [15]. This technique typically yields a neutrophil purity of around 90% [16]. For comparison, peripheral blood neutrophils were isolated from the same subjects on the day of cell harvest. Secondly, after written informed consent, synovial fluid cells were isolated from the knee joints of three patients with inflammatory arthritis during a disease flare (one polyarthritis, one rheumatoid arthritis and one spondylarthritis). Synovial fluid was aspirated, and within 2 h, the fluid was filtered through a cell strainer and centrifuged before resuspension of the cells in KRG and storage on ice until use. The synovial fluids included in the study contained between 80-90% neutrophils, as determined by flow cytometry on live cells. Peripheral blood was drawn and neutrophils isolated from the same study subjects on the day of synovial fluid aspiration.

### Immunofluorescent staining

OLFM4 was stained using indirect immunofluorescence. Neutrophils in suspension (1x10^6^ per sample), neutrophil cytospins on microscope slides (2x10^5^), or NETs on glass cover slips (see below) were fixed using 4% paraformaldehyde, washed, and permeabilized using ice cold acetone and methanol at a 1:1 ratio for 5 min, or left unpermeabilized as indicated. Unspecific staining was blocked for using blocking buffer (10% normal goat serum and 2% bovine serum albumin). Polyclonal rabbit anti-human OLFM4 antibody (Abcam, Cambridge, UK, 5 µg/ml) was added and samples incubated for 30 min. After washing, samples were incubated with Alexa Fluor 488- or 647-conjugated goat anti-rabbit IgG (F(ab’)_2_ fragment) (Invitrogen, Carlsbad, CA, USA, 5 µg/ml) for 30 min, and washed. Samples in suspension were then analyzed using a flow cytometer (BD Accuri C6, data analyzed in CFlow software and displayed using FlowJo). Alternatively, samples were supplemented with DAPI at 60 nM for staining of DNA and analyzed using an imaging flow cytometer (Amnis ImageStream^10^, data analyzed using the colocalization wizard in Ideas software v. 5.0 as described by others [17]). Samples for microscopy were mounted using ProLong Antifade with DAPI (Invitrogen) and then analyzed using a confocal microscope (Zeiss LSM 700 equipped with Zen 2009 software for acquiring images that were then processed in PhotoShop CS5.1). Isotype controls, or the omission of primary antibody, were routinely used to ensure specific binding of the antibodies, and to set gates for positive staining.

Other granular proteins were stained using the same procedure. Mouse monoclonal anti-human neutrophil gelatinase associated lipocalin (NGAL) antibody (Abcam) was diluted to 5 µg/ml, mouse monoclonal anti-human myeloperoxidase (MPO) antibody (DAKO, Glostrup, Denmark) was diluted to 200 µg/ml, mouse monoclonal anti-human CD63 antibody (Sanquin, Amsterdam, The Netherlands) was diluted to 2 µg/ml, and rabbit anti-human lactoferrin antibody (DAKO) was diluted to 100 µg/ml. Alexa Fluor 488- or 647-conjugated secondary antibodies were used in these experiments. CD177 was stained using a mouse anti-human CD177 antibody conjugated with phycoerythrin (Abcam), diluted 1:5, and CD16 with a mouse monoclonal anti-human CD16 antibody conjugated with fluorescein isothiocyanate (FITC) (EuroBioSciences, Friesoythe, Germany), diluted 1:20. Single stains were routinely performed to rule out overbleed between fluorescence channels in microscopy or compensate for these in flow cytometry and imaging flow cytometry.

### Subcellular fractionation and Western blot

Subcellular fractionation of disintegrated neutrophils was performed on discontinuous Percoll gradients by the Borregaard [18] technique. Briefly, diisopropyl fluorophosphate- (Fluka, Seelze, Germany) treated neutrophils were disrupted by nitrogen cavitation (Parr Instrument Company, Moline, IL, USA), after which intact cells and nuclei were removed by a low speed centrifugation. The postnuclear supernatant was layered onto a three step Percoll gradient and after centrifugation (17000 x *g* for 30 min), 1 mL fractions were collected from the bottom of the centrifuge tube. Granule marker analysis was performed to ensure separation in the gradient of the following organelles: azurophil granules (MPO), specific granules (lactoferrin), geleatinase granules (gelatinase), secretory vesicles (latent alkaline phosphatase) and plasma membranes (alkaline phosphatase). The contents of lactoferrin and gelatinase were analyzed by Western blotting, using rabbit anti-human lactoferrin (DAKO) and rabbit anti-human gelatinase (Merck, Darmstadt, Germany), both at 2 µg/ml. The subcellular localization of OLFM4 was also determined by Western blotting, using polyclonal rabbit anti-human OLFM4 antibody (Abcam, 0.5 µg/ml).

### Conventional degranulation assay

In order to induce different degrees of secretion of granule contents, two secretagogic stimulations were used. Neutrophils diluted in KRG were treated at 37^o^C with cytochalasin B (5 µg/ml) for 5 min followed by ionomycin (0.5 µM) for 10 min, a strong non-physiological stimulation that results in efficient release of specific granules [19]. Alternatively, the cells were incubated at 15^o^C without stimulus followed by 10 min at 37^o^C with the addition of N-formyl-Met-Leu–Phe (fMLF, 10^-7^ M), a physiological, weaker stimulation that causes release of approximately 10% of specific granules [20]. Subsequently, fixation and staining of cells was carried out as described above. Fluorescence intensity in the isotype controls did not change with stimulation (data not shown).

### Apoptosis induction and detection

Neutrophils were allowed to enter apoptosis spontaneously or in the presence of Fas ligand (FasL) at 10 µg/ml (mouse anti-human CD95, eBiosciences, San Diego, CA, USA), essentially as described [21], for either 4 h or 20 h at 37^o^C with 5% CO_2_. The cells were then either fixed and stained for OLFM4 as described above or left non-fixed. To detect apoptosis in fixed and stained cells, a TdT-mediated dUTP-X nick end labeling (TUNEL) assay (In Situ Cell Death Detection Kit, Fluorescein, Roche, Penzberg, Germany) was used to detect fragmented DNA, according to the manufacturer’s instructions, and analyzed by flow cytometry. To study cell death in non-fixed cells incubated in the presence or absence of recombinant OLFM4 (rOLFM4, 0.1, 0.5, or 1 µg/ml), neutrophils were washed and stained using Annexin V-Fluos (for apoptosis) and 7-AAD (for necrosis), and analyzed using flow cytometry as previously described in [22].

### Phagocytosis assay

The avirulent *Mycobacterium tuberculosis* laboratory strain H37Ra expressing GFP was used in phagocytosis assays. The bacteria were cultured and prepared for infection as previously described [23], and then either complement opsonized through incubation for 30 min at 37^o^C in 50% normal human serum, or left unopsonized. Neutrophils were diluted in KRG to 5x10^6^/ml, and without further washing of the bacteria, phagocytosis was carried out for 30 min at 37^o^C at a multiplicity of infection of 5:1. Fixation and staining of OLFM4 was performed as described above, and samples were analyzed by flow cytometry.

### Induction of NET formation

NET formation was induced essentially as described in [24]. Briefly, neutrophils isolated from whole blood were diluted to 4x10^5^/ml in RPMI supplemented with 2% fetal calf serum and seeded on glass cover slips placed in a 24-well plate (500 µl/well), and allowed to adhere for 1 h at 37^o^C with 5% CO_2_. They were then stimulated with phorbol myristate acetate (PMA, 20 nM) for 3 h at 37^o^C with 5% CO_2,_ fixed, and processed for immunofluorescent staining as described above. In order to visualize the plasma membranes of the cells, the stained samples were incubated with FITC-conjugated wheat germ agglutinin (WGA, 1 µg/ml), which is a sugar-binding protein with specific plasma membrane affinity [25], for 10 min at RT, and washed three additional times prior to mounting in DAPI-containing medium as above. Confocal microscopy was performed as described above.

### Neutrophil activation assay

The shedding of L-selectin from the cells is indicative of neutrophil activation. Neutrophils were treated with TNFɑ (10 µg/ml), rOLFM4 (1 µg/ml), or left untreated for 20 min at 37^o^C. They were then washed and stained for L-selectin using PE-conjugated mouse anti-human CD62L (BD Biosciences, Franklin Lakes, NJ, USA) diluted 1:40, as previously described in [16]. The mean fluorescence intensity of L-selectin staining was measured using flow cytometry.

## Results

### OLFM4 is a specific granule protein expressed in a subset of human neutrophils in circulation

**Figure 1:**
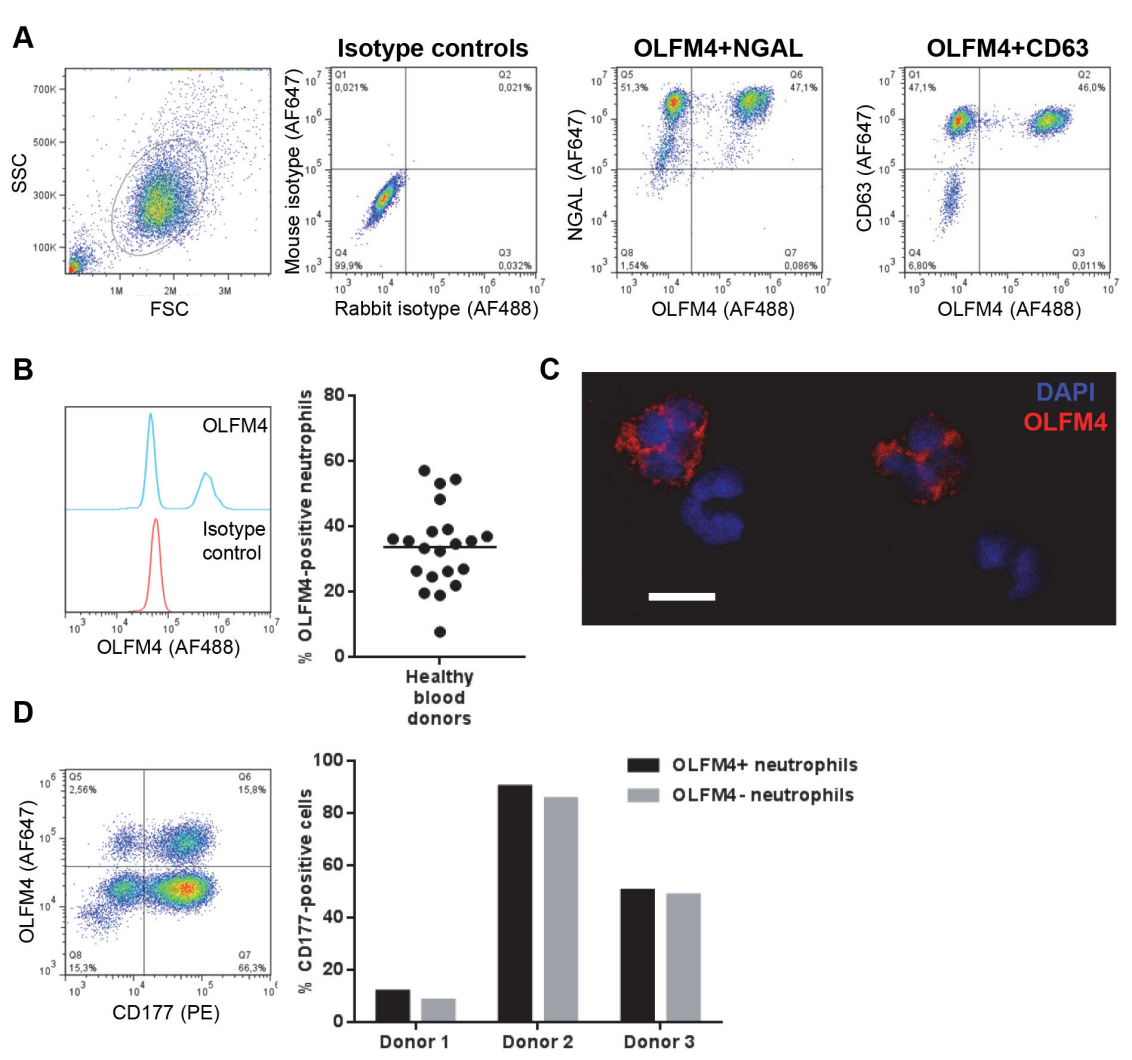
Analysis of OLFM4 expression in human neutrophils. **A**) Immunostained neutrophils were analyzed by flow cytometry. The first dot plot shows forward versus side scatter and the neutrophil gate in a representative donor. The two subsequent dot plots show double staining of isotype controls, OLFM4 and NGAL, or OLFM4 and CD63 in one representative experiment out of five. **B**) OLFM4 was stained in neutrophils isolated from 21 individuals. The histogram shows OLFM4 (blue) and isotype control (red) staining in neutrophils from one representative donor. The diagram shows the percentage of OLFM4-positive neutrophils in the individual donors, and the line shows the mean. **C**) Confocal image showing OLFM4 (red, Alexa Fluor 647) and DNA (DAPI, blue) in neutrophils attached to microscope slides. Four individual cells from one field of vision are shown. The scale bar represents 5 µm. **D**) Co-staining of OLFM4 and CD177. The figure shows a representative dot plot of the double staining, with the quadrants set using isotype controls. The bar graph depicts the percentage of CD177-positive neutrophils in the OLFM4-positive (black) and -negative (grey) subpopulations in three individual donors.

In order to study the expression of OLFM4 protein in human neutrophils in circulation, we isolated and fixed neutrophils from peripheral blood of healthy volunteers. The cells were permeabilized and immunofluorescently stained for OLFM4 together with other known granule markers. Typically around 95% of the gated cells stained positively for NGAL, a specific granule matrix protein, as well as for CD63, a protein present in the membrane of azurophil granules, while only a clearly distinct subpopulation stained positively for OLFM4 (Figure 1A). Isolated neutrophils from 21 independent whole blood donors were stained for OLFM4 and the OLFM4-positive proportion varied between 8% and 57%, with a mean of 34% (Figure 1B). To visualize the two OLFM4-defined neutrophil subpopulations, we immunostained for OLFM4 in neutrophils adhered to microscope slides, and again confirmed the presence of clearly distinguishable OLFM4-positive and -negative subpopulations (Figure 1C).

It is noteworthy that the method used for permeabilization of the cells was essential to capture OLFM4-positivity, and saponin permeabilization was insufficient for efficient staining of OLFM4 (data not shown). Using acetone and methanol for permeabilization, OLFM4 expression in one individual was similar over time (range of 22-26% OLFM4-positive neutrophils at three different sampling times over the course of one year), and parallel staining of neutrophils from different donors gave different OLFM4-positive proportions. Isolated peripheral blood mononuclear cells showed very low expression of OLFM4 in all cells, and purified eosinophils showed no expression of OLFM4 (data not shown). Furthermore, we confirmed the existence of two subpopulations using commercially available anti-neutrophil cytoplasmic antibody (ANCA) slides containing ethanol-fixed human neutrophils, yielding a cytoplasmic ANCA staining pattern in only a subpopulation of the cells (data not shown), providing an external control of our protocol. Finally, the OLFM4-negative and OLFM4-positive neutrophil subsets could not only be detected in isolated cells, but also in whole blood, after lysis of erythrocytes and staining of all leukocytes (data not shown).

Aside from OLFM4, neutrophil subpopulations defined by the presence or absence of CD177 have been described. This molecule is present on the plasma membrane and in granules of 0 to 100 % of neutrophils in healthy individuals [6,26]. Double staining of neutrophils for OLFM4 and CD177 revealed that CD177 expression was distributed evenly among the subsets defined by OLFM4 (Figure 1D), showing that the markers do not co-vary.

**Figure 2:**
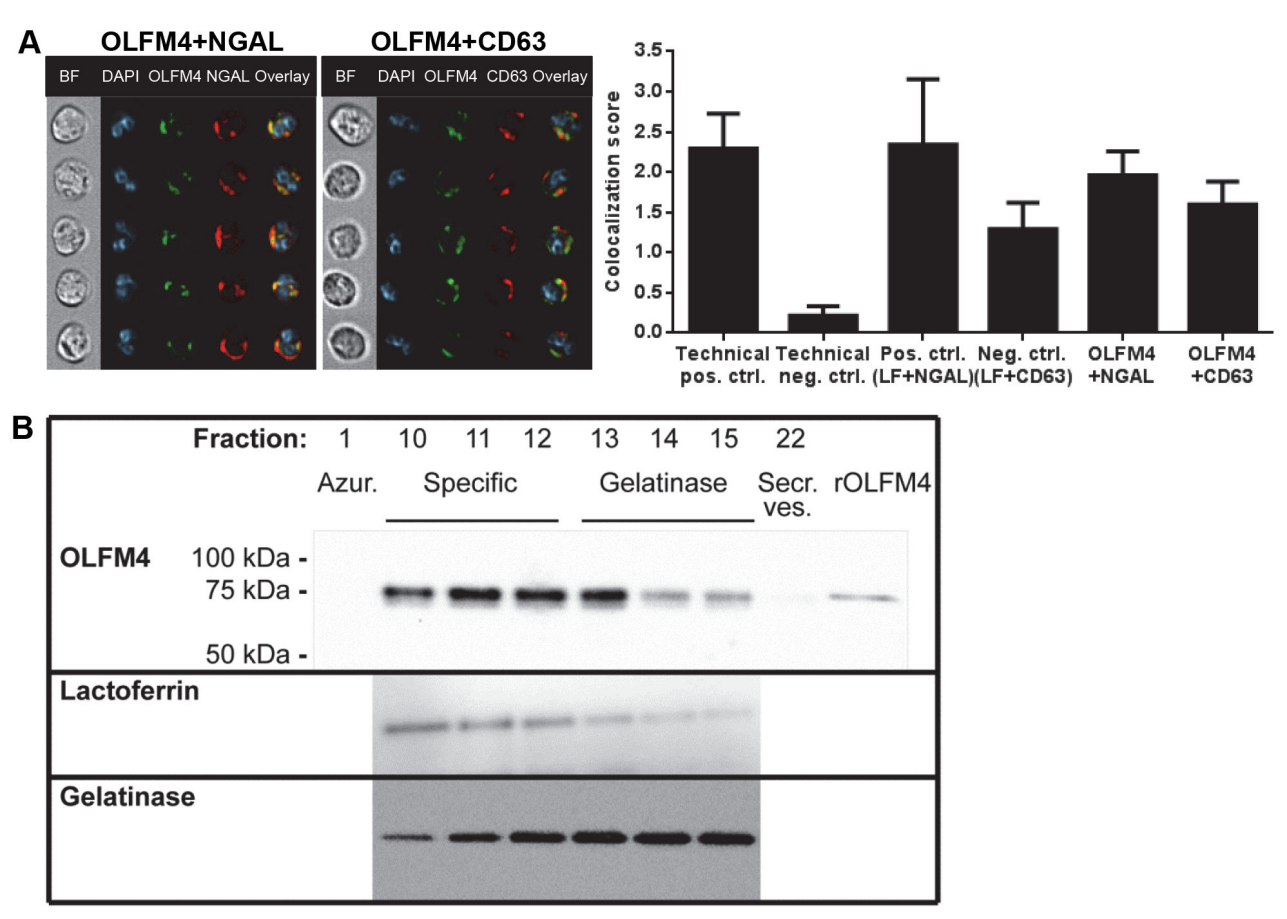
Subcellular localization of OLFM4. **A**) Neutrophils were immunofluorescently stained for OLFM4 (green) together with specific granule marker NGAL or azurophil granule marker CD63 (red), and DNA was labeled with DAPI (blue). Colocalization was analyzed by imaging flow cytometry and images show representative cells from the double positive populations (BF = brightfield). The diagram shows the mean colocalization score for the two fluorophores +SD from at least three experiments. The technical positive control was FITC-conjugated mouse anti-human CD16 antibody followed by Alexa Fluor 647-coupled goat anti-mouse secondary antibody and the technical negative control was FITC-conjugated mouse anti-human CD16 antibody together with DAPI, showing the minimum and maximum values that can be obtained by this analysis. The biological positive control was specific granule marker lactoferrin (LF) together with NGAL, and the biological negative control was lactoferrin together with CD63, showing the resolution of the analysis. **B**) Pooled neutrophils from three donors were subjected to subcellular fractionation, and the fractions containing peak content of azurophil granule marker (MPO; fraction 1), specific granule marker (lactoferrin; fractions 10-12), gelatinase granule marker (gelatinase; fractions 13-15) and secretory vesicle marker (latent alkaline phosphatase; fraction 22) were subjected to Western blot with anti-OLFM4 antibody using rOLFM4 as a positive control. One representative blot out of three is shown. Lactoferrin (specific granule marker) and gelatinase (gelatinase granule marker) blots are also shown for fractions 10-15.

OLFM4 has been found by others to be expressed mainly in the specific granules of neutrophils [7,10]. In order to confirm this, we first employed an imaging flow cytometry method followed by colocalization analysis of neutrophils immunofluorescently stained for OLFM4 and the specific granule marker NGAL. This revealed a high degree of colocalization between OLFM4 and NGAL, while that between OLFM4 and the azurophil granule marker CD63 was lower, consistent with specific granule localization (Figure 2A). We supplemented these findings with an investigation of OLFM4 protein abundance in different granule fractions after subcellular fractionation. The azurophil granule fraction tested (fraction 1, identified by MPO content) and secretory vesicle fraction tested (fraction 22, identified by latent alkaline phosphatase activity) did not contain OLFM4 (Figure 2B). A clean distinction between specific granules (identified by lactoferrin) and gelatinase granules (identified by gelatinase) is not possible using this method, but the OLFM4 staining pattern was very similar to that of lactoferrin, again indicating mainly specific granule localization (Figure 2B). In fact, the different granule subsets are not entirely distinct from each other, but rather form a continuous gradient where different cargo is packed into the granules depending on the time of formation during myelopoeisis, a mechanism termed “targeting by timing of biosynthesis” [1].

### OLFM4 expression does not predispose neutrophils to apoptosis or phagocytosis

**Figure 3:**
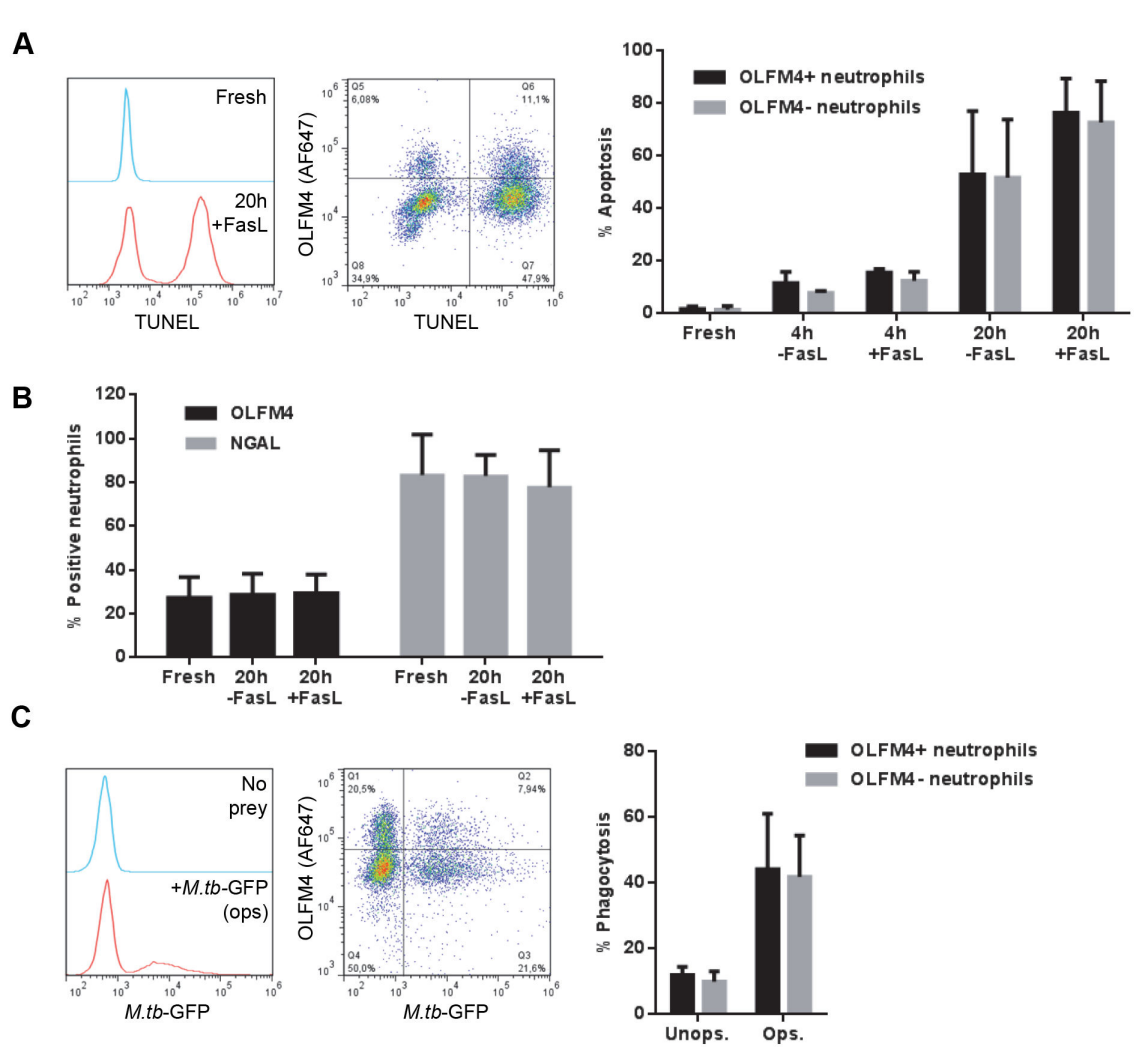
Functional analyses of the neutrophil subpopulations. **A**) Neutrophils were either fixed immediately after separation (Fresh) or allowed to enter apoptosis spontaneously (-FasL) or through the Fas pathway (+FasL) for 4 or 20 h, after which they were fixed and stained using a TUNEL assay in combination with immunostaining for OLFM4. The histogram shows fresh (blue) and apoptotic (20 h +FasL, red) neutrophils stained using TUNEL. The dot plot shows double staining of fragmented DNA (TUNEL) and OLFM4, with the quadrants set using fresh neutrophils for TUNEL and control (omitted primary antibody) for OLFM4. The bar graph shows the mean percentage of apoptosis +SD, based on TUNEL-positivity, in the OLFM4-positive (black) and -negative (grey) subpopulations of three donors. **B**) Freshly isolated and apoptotic neutrophils (20 h incubation without or with FasL) were subjected to immunostaining of OLFM4 (black) and NGAL (grey). The diagram shows the mean percentage of positive cells +SD for each protein from three independent experiments. **C**) Neutrophils were allowed to phagocytose *M. tuberculosis* H37Ra strain expressing GFP (*M*.*tb*-GFP), either unopsonized or serum-opsonized, at an MOI of 5 for 30 min, fixed, and immunostained for OLFM4. The histogram shows neutrophils incubated without (No prey) or with (+*M*.*tb*-GFP ops) serum-opsinized *M. tuberculosis*. The dot plot shows fluorescence intensity in the GFP and OLFM4 channels after phagocytosis of opsonized bacteria, with the quadrants set using neutrophils with no prey added for *M*.*tb*-GFP and control (omitted primary antibody) for OLFM4. The bar graph shows the mean percentage of phagocytosing neutrophils +SD in the OLFM4-positive (black) and -negative (grey) subpopulations from three independent experiments.

Having confirmed the existence of an OLFM4-positive and an OLFM4-negative neutrophil subpopulation in human blood, we wanted to investigate whether the subsets are functionally different. First, we tested whether the proneness to undergo apoptosis differed between the subsets by incubating neutrophils for 4 or 20 h with or without FasL, an effective inducer of apoptosis, after which cells were stained for fragmented DNA (a sign of apoptosis) using TUNEL, together with OLFM4. The FasL-treatment resulted in varying degrees of cell death, but OLFM4-positive and -negative neutrophils were equally prone to undergo apoptosis (Figure 3A). Furthermore, quantification of OLFM4- and NGAL-positivity in freshly prepared cells compared to cells subjected to overnight incubation with or without FasL showed that OLFM4-positivity does not change with cell age or induction of apoptosis (Figure 3B). This is consistent with Clemmensen et al. [7], who found that bimodal expression of OLFM4 appears at the myelocyte stage of maturation in the bone marrow, indicating that loss or gain of OLFM4 is not a function of cell age.

To investigate another functional aspect that could potentially differ between subsets of neutrophils, we chose to study phagocytosis in the OLFM4-positive and -negative populations using *M. tuberculosis* as a model organism. However, no difference in the proportion of phagocytosing cells was found between the two subpopulations, regardless of whether unopsonized or opsonized prey was used (Figure 3C). The same result was obtained when unopsonized or serum-opsonized yeast was used as prey (data not shown).

### The OLFM4-expressing subpopulation transmigrates into tissue

**Figure 4:**
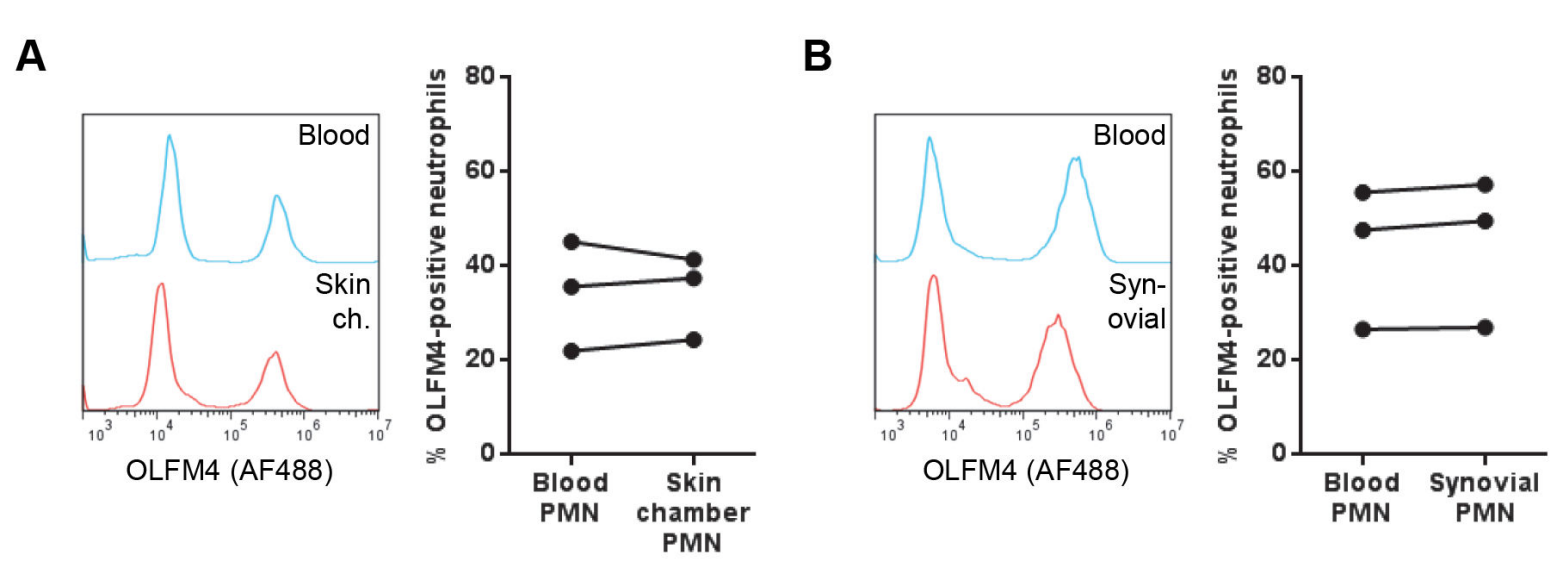
OLFM4 expression in transmigrated tissue neutrophils. Skin chamber neutrophils (**A**) were obtained from healthy volunteers and synovial fluid neutrophils (**B**) were collected from inflammatory arthritis patients. Blood samples were also drawn from all subjects. After fixation of tissue and blood neutrophils, OLFM4 was immunolabelled. The histograms show OLFM4 staining in blood and skin chamber (Skin ch.) or synovial fluid neutrophils from one donor each. The diagrams show the percentage of OLFM4-positive neutrophils in blood and transmigrated neutrophils from three different donors.

As neutrophils typically carry out their major functions after transmigration from the bloodstream, it is highly relevant to ask whether both neutrophil subsets are able to transmigrate to extravascular tissue. A potential difference in the ability to transmigrate could be an effect of OLFM4 itself, or more likely, of other factors that characterize the OLFM4-defined subsets. We investigated this using cells obtained in two different manners. Firstly, we used experimental skin chambers, an established controlled model of *in vivo* transmigration to acute inflammation in otherwise healthy skin [15]. By comparing OLFM4-positivity in skin chamber- and blood neutrophils from the same donor, we found that the OLFM4-positive and –negative subpopulations both indeed transmigrated into tissue, and to an equal extent (Figure 4A). Secondly, we obtained *in vivo* transmigrated neutrophils from naturally occurring, pathologically inflamed tissue by aspirating synovial fluid from the knee joints of patients with inflammatory arthritis. Again, when comparing the tissue neutrophils to blood neutrophils from the same subjects, we found that the proportion of OLFM4-positive cells was similar in blood and tissue (Figure 4B). In addition to demonstrating the ability of both subsets to transmigrate, these results indicate that OLFM4 is largely retained inside the cell after *in vivo* transmigration. Of note, the proportion of OLFM4-positive blood neutrophils did not differ markedly between inflammatory arthritis patients and healthy controls (compare blood neutrophils in Figure 4A and 4B).

### Conventional secretion of OLFM4 is limited under physiological conditions

**Figure 5:**
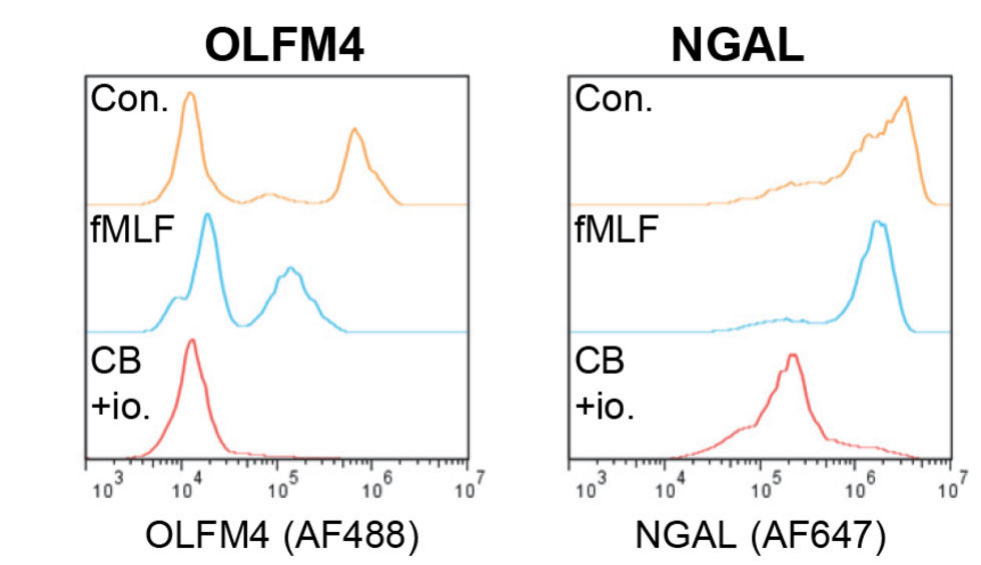
Conventional degranulation of OLFM4. Control neutrophils and neutrophils treated with fMLF or cytochalasin B/ionomycin were subjected to immunofluorescent staining of OLFM4 and NGAL, and analyzed by flow cytometry. The histograms show OLFM4 or NGAL staining in control (Con, yellow), fMLF-stimulated (blue), and cytochalasin B/ionomycin-stimulated (CB+io, red) neutrophils from one representative experiment out of four.

The fact that the OLFM4-positive subpopulation remained clearly discernible after *in vivo* transmigration into inflamed tissue is consistent with specific granule localization, as specific granules are only marginally secreted during transmigration [27]. For degranulation of specific granules to occur, strong secretagogic stimulation is required [19,20,28]. In line with this, after stimulation of blood neutrophils with a weak, physiological secretagogue (fMLF [20]), most OLFM4 was retained with a slight uniform decrease in fluorescence intensity of the OLFM4-positive subpopulation (Figure 5). In contrast, strong secretagogue stimulation (cytochalasin B + ionomycin [19]) caused the OLFM4-positive subpopulation to disappear altogether, indicative of complete release of OLFM4 from the cells (Figure 5). This is again consistent with specific granule localization of OLFM4, and NGAL showed a very similar pattern (Figure 5).

### OLFM4 is a constituent of NETs formed from OLFM4-positive neutrophils

**Figure 6:**
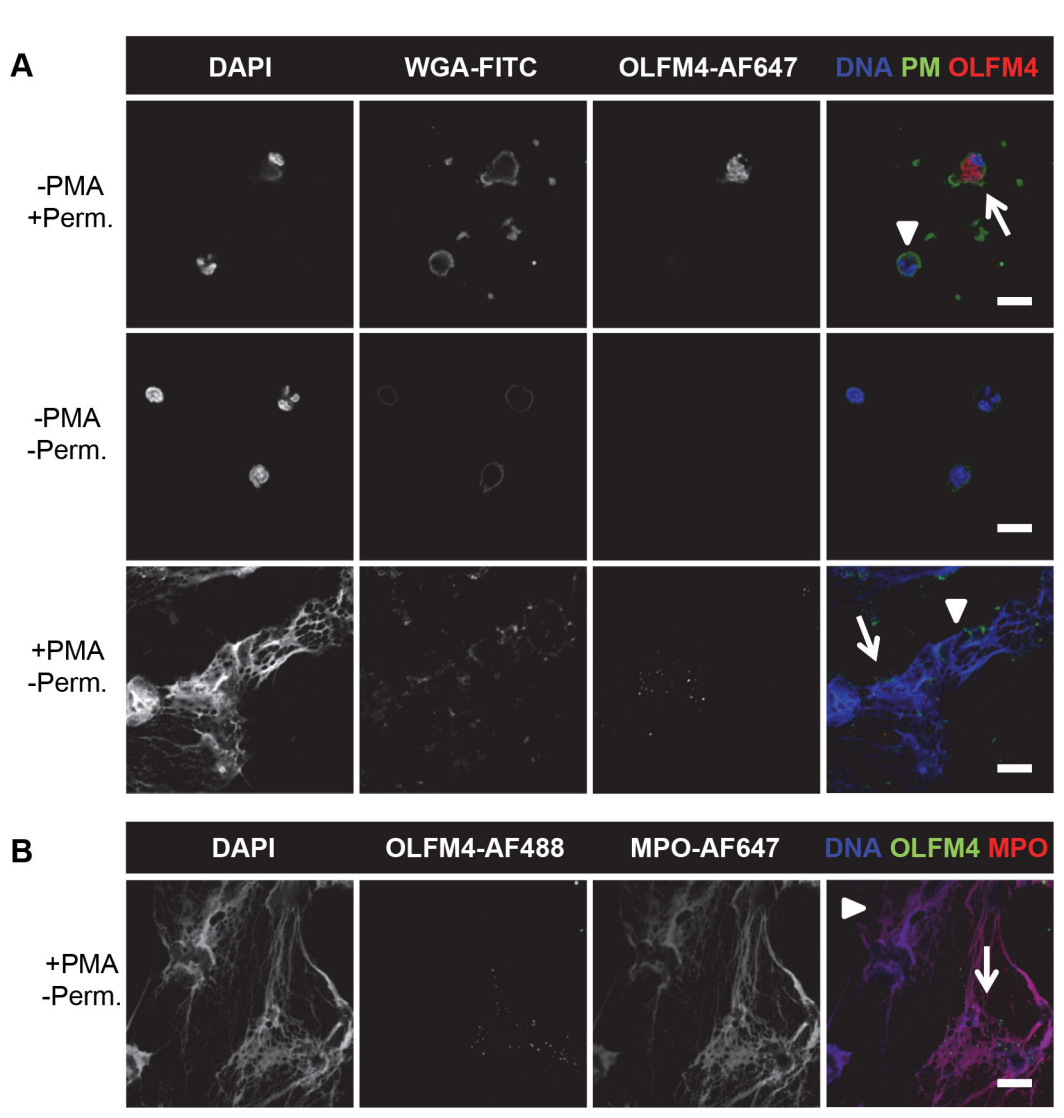
Presence of OLFM4 in NETs. **A**) Neutrophils isolated from heparinized blood were adhered to glass coverslips and either stimulated with PMA to form NETs (+PMA) or left unstimulated (-PMA). They were then left unpermeabilized (-Perm.) or permeabilized using acetone/methanol (+Perm.), after which they were immunostained for OLFM4 (red), plasma membranes (PM) were stained using FITC-conjugated WGA (green), and DNA was stained with DAPI (blue). Confocal images show representative cells or NETs from at least three individual experiments. **B**) Adherent neutrophils were induced to form NETs as in A, and unpermeabilized samples were immunostained for OLFM4 (green) and MPO (red). DNA was stained with DAPI (blue). Confocal images show representative NETs from three individual experiments. **A**–**B**: Arrows indicate OLFM4-containing cells or NETs, while arrow heads indicate cells or NETs without OLFM4. The fluorophore conjugates used for each staining are indicated (AF = Alexa Fluor). The scale bars represent 5 µm.

Since physiological stimulation (*in vivo* as well as *in vitro*) did not lead to major release of OLFM4, we wondered whether non-conventional mechanisms of secretion could be a route to release OLFM4. We postulated that NET formation may be one situation where the OLFM4 protein can be exposed extracellularly, with the potential to affect surrounding immune components. NETs are extracellular meshwork-like structures composed of processed chromatin, granular proteins and selected cytosolic proteins, and are thought to function to trap microbes [12,29], but also to play a role in autoimmune disorders by exposing otherwise intracellular endogenous components to the immune system [30–32]. Whereas acetone/methanol permeabilization was required to detect OLFM4 in unstimulated adherent cells (Figure 6A) the protein was readily detected without permeabilization after induction of NET formation (Figure 6A). Interestingly, OLFM4 was only found in a subpopulation of the NETs, likely those arising from the OLFM4-positive subpopulation. We indirectly tested this by incubating either unfixed or fixed NETs with rOLFM4 prior to staining as described above. OLFM4 did not bind DNA under the conditions tested, as the staining pattern looked identical in NETs incubated with or without rOLFM4 (data not shown). These results together suggest that the OLFM4 found in a NET originates from the neutrophil that formed the NET, rather than having been released and then bound back to the DNA. In order to further confirm the localization of OLFM4 to only a subpopulation of NETs, we performed double staining of OLFM4 and the known granule-derived NET protein MPO [33]. MPO could be found uniformly in all NETs while OLFM4 was only found in a subset (Figure 6B). OLFM4 staining in NETs was not due to unspecific binding of the secondary antibody (Figure S1). Thus, during NET formation, OLFM4 localizes to the NET of its parent cell. To our knowledge, this is the first report of two different subtypes of NETs.

**Figure 7:**
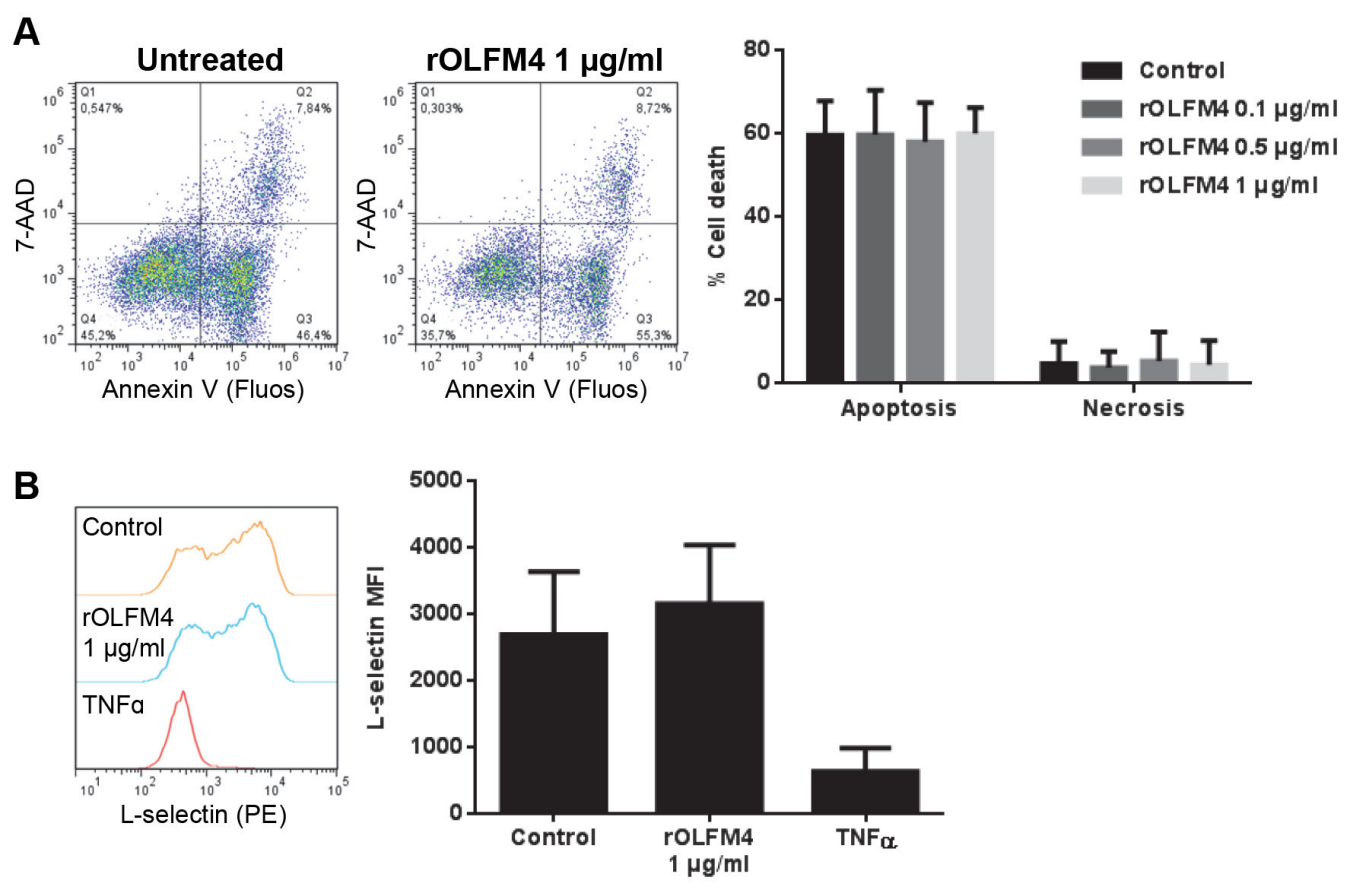
Effect of rOLFM4 protein on neutrophils. **A**) Neutrophils were allowed to enter apoptosis spontaneously (untreated) or in the presence of rOLFM4 at a range of concentrations, after which they were stained using Annexin V-Fluos (for apoptosis) and 7-AAD (for necrosis). The dot plots show Annexin V and 7-AAD staining in untreated and rOLFM4-treated cells in one representative donor after 20 h incubation, and the histogram shows the percentage of neutrophils that have undergone each type of cell death in untreated and rOLFM4-treated samples, respectively. The mean +SD of three experiments is shown. **B**) Neutrophils were treated with rOLFM4 (1 µg/ml) , TNFɑ (10 µg/ml; positive control) or left untreated, for 20 min at 37^o^C, and analyzed for surface expression of L-selectin. The shedding of L-selectin from the cells is indicative of neutrophil activation. The histogram shows L-selectin staining in untreated cells and cells treated with rOLFM4 or TNFɑ in one representative experiment. The bar graph shows the mean fluorescence intensity (MFI) of L-selectin +SD from three independent experiments.

Release of OLFM4 into NETs indicates that surrounding cells as well as invading microbes can be exposed to and affected by the protein. The gradual loss of L-selectin from neutrophils is indicative of cell activation, with resting cells uniformly displaying high L-selectin staining, cells treated with TNF-α at 37^o^C displaying low staining, and untreated cells incubated at 37^o^C displaying intermediate staining [34,35]. Adding rOLFM4 to neutrophils did not activate the cells to shed L-selectin as compared to the control (Figure 7A). Nor did rOLFM4 alter neutrophil viability after 20h in culture (Figure 7B). We also did not find any direct effect of rOLFM4 (at a concentration of 20 µg/ml) on the growth of *E. coli* or *S. epidermidis* (data not shown), using an inhibition zone assay as previously described [36]. 

## Discussion

In the 1980’s, Gallin [3] established that neutrophil heterogeneity existed, and debated whether the phenomenon had any biological relevance. Several of the markers discussed by Gallin to be differentially expressed on the neutrophil cell surface [3] can be explained by different degree of neutrophil activation, and degranulation in the systems studied. This is also true for several of the different neutrophil subsets more recently discovered to be associated with systemic inflammation [37,38], and with tumours and their promotion or suppression [39,40]. Prominent examples of subset markers that change with cell activation include L-selectin and CD11b [37,38], which are present on all neutrophils but differentially displayed depending on the degree of cell activation [2,34,35]. However, also more recently, neutrophil heterogeneity in terms of bona fide protein expression has been described [4,5]. The latter subpopulation markers are more robust, with a genetic basis in the case of CD177 [26] and an unknown post-transcriptional mechanism being responsible in the case of OLFM4 [7]. These recent findings prompted us to revisit the intriguing question posed by Gallin with today’s platform of knowledge and analytical tools, using the two subpopulations defined by the presence or absence of OLFM4 [7] as a foundation.

We first confirmed that two neutrophil subsets, one expressing OLFM4 and one not, are present among human peripheral blood neutrophils [7], and that OLFM4 is a specific granule protein [7,10]. The higher average proportion of OLFM4-positive cells in our study as compared to that found by Clemmensen et al. [7] is likely due to donor variability.

We then set out to study whether any functional differences between the subsets could be demonstrated. First, we found that the OLFM4-positive and -negative subpopulations underwent apoptosis at the same rate. Similarly, there was no difference in phagocytic ability between the subsets. Although it has not been investigated whether OLFM4-defined subpopulations are present in mice, our data agree with murine studies comparing OLFM4 knockout with wild type mice that argue for a negative role of OLFM4 during bacterial infection, despite similar levels of neutrophil phagocytosis [10,11]. A third functional aspect that we speculated might differ between the neutrophil subsets was the ability to transmigrate into tissue. However, comparison of blood and tissue neutrophils, obtained from a chronically inflamed site (synovial fluid neutrophils) and a site of experimentally induced acute inflammation (skin chamber neutrophils), revealed that blood and tissue neutrophils from the same donor had equal proportions of OLFM4-positive cells. This indicates the same propensity of the subpopulations to transmigrate into tissue, and that there is no difference between the subsets in terms of recruitment or chemotaxis. A functional difference between the two subsets remains to be found. Such a difference, if present, may not necessarily be a direct effect of OLFM4 *per se*, but rather a function of other factors that possibly co-vary with OLFM4 due to co-regulation.

Our results show that the OLFM4-defined neutrophil subsets are also present in tissue where neutrophils typically carry out their main tasks, and they are not merely a phenomenon in circulation. Tissue neutrophils are generally more activated than those in circulation [34], and *in vivo* transmigration leads to the mobilization of most gelatinase and a small proportion of specific granules [1]. The similarity in OLFM4-positivity that we observed in blood and tissue is consistent with specific granule localization, as specific granules are largely retained inside the cells during transmigration [1,27]. Our data furthermore show that differential OLFM4-expression is not dependent on neutrophil stimulation or related to degree of neutrophil activation, in contrast to several other recently described neutrophil subpopulation markers [5,37–40].

Having found that both OLFM4-positive and –negative neutrophils are recruited to sites of inflammation, we wanted to determine whether the OLFM4 protein itself could be exposed to the extracellular environment. Secretagogic stimulation with the weak, physiological fMLF led to uniform but only partial release of OLFM4, while the non-physiological, strong cytochalasin B + ionomycin treatment caused complete release. Again, this is consistent with specific granule localization, and our finding corroborates that of Clemmensen et al. [7] who used PMA stimulation, which gives efficient release of about 50% of the specific granules [28], and obtained a reduction of OLFM4 in specific granule fractions. Our results indicate that only low levels of OLFM4 are released during transmigration, even to pathologically inflamed sites, and that conventional degranulation is not a major physiological source of extracellular OLFM4.

Neutrophils have multiple roles in tissue during infection. These include phagocytosis and intracellular killing of microbes, as well as release of antimicrobial substances into the extracellular space through degranulation, and the formation of NETs. NETs are networks of DNA and granular proteins that are thought to trap and kill microorganisms [12,29], but can also be thought of as an alternative means of secretion since their formation inevitably leads to the exposure of intracellular content to the extracellular space. NETs have been shown to form both *in vitro* in response to certain stimuli [12] and *in vivo* in tissue [41]. As we found that conventional degranulation did not lead to major OLFM4 release, we instead turned to NETs and hypothesized that NET formation could lead to OLFM4 secretion. Our data show that upon the formation of NETs through a standard protocol that employs extensive PMA stimulation of adherent cells, extracellular OLFM4 can indeed be detected in the NETs. The presence of OLFM4 in NETs was recently described using a proteomics approach, supporting our finding [42]. OLFM4 was found only in a subpopulation of the NETs, while MPO was found in all NETs, indicating that two OLFM4-defined subsets of NETs exist. Our results indicate that during NET formation, OLFM4 is expelled from each respective cell containing the protein into the NET originating from that cell, rather than being released into the medium and then binding to all DNA. This notion supports the theory that granular proteins associate with DNA prior to expulsion from the cell during NET formation [12].

Although our experiments did not indicate a direct effect of rOLFM4 on neutrophil activation or viability, nor a direct antibacterial effect (in agreement with Liu et al. [10]), exposure of OLFM4 in the extracellular space through NETs may still have a profound effect on the immune system. Analogously, CD177-positive neutrophils display proteinase 3 (PR3) on their surface and are thus a target of ANCA, present in some autoimmune disorders [43,44]. Binding of ANCA to CD177 positive (and thereby PR3 positive) cells activates them [45], and it has been proposed that having a high proportion of CD177-positive neutrophils confers an increased risk of developing vasculitis [46]. Several recent studies have found that antigens present in NETs, such as DNA [30], histones [31], MPO or PR3 [32] can give rise to clinically relevant autoantibodies in different disorders. It remains to be shown whether ANCA directed against OLFM4 exist and have clinical implications.

In conclusion, we confirm the existence of two OLFM4-defined neutrophil subsets in human circulation and furthermore show that both subsets are capable of transmigration into tissue. OLFM4 can be found in a subpopulation of NETs, suggesting the existence of two distinct NET populations. Even though the underlying mechanisms to explain OLFM4 heterogeneity are unknown, the simultaneous existence of numerous neutrophil subtypes is a fact; interestingly, co-staining of OLFM4 and CD177 actually revealed four distinct subpopulations. As of yet, functional studies have provided no evidence that neutrophil heterogeneity is biologically relevant, but as evolution rarely fosters the development of meaningless traits, the phenomenon deserves further investigation. 

## Supporting Information

Figure S1 Specificity of OLFM4 staining in NETs. Neutrophils isolated from heparinized blood were adhered to glass coverslips and stimulated with PMA to form NETs. Unpermeabilized samples were immunostained for OLFM4 (green) and MPO (red) (top panel). Alternatively, isotype controls followed by secondary antibody was used (bottom panel). DNA was stained with DAPI (blue). The arrow indicates OLFM4-containing NETs, while the arrow head indicates NETs without OLFM4. The fluorophore conjugates used for each staining are indicated (AF = Alexa Fluor). The scale bars represent 5 µm.
(TIF)
