# Supplementary figures and images for: The Human Neutrophil Subsets Defined by the Presence or Absence of OLFM4 Both Transmigrate into Tissue In Vivo and Give Rise to Distinct NETs In Vitro
Source: PLoS One. 2013 Jul 29;8(7):e69575. doi: 10.1371/journal.pone.0069575 (PMC3726694; doi:10.1371/journal.pone.0069575)

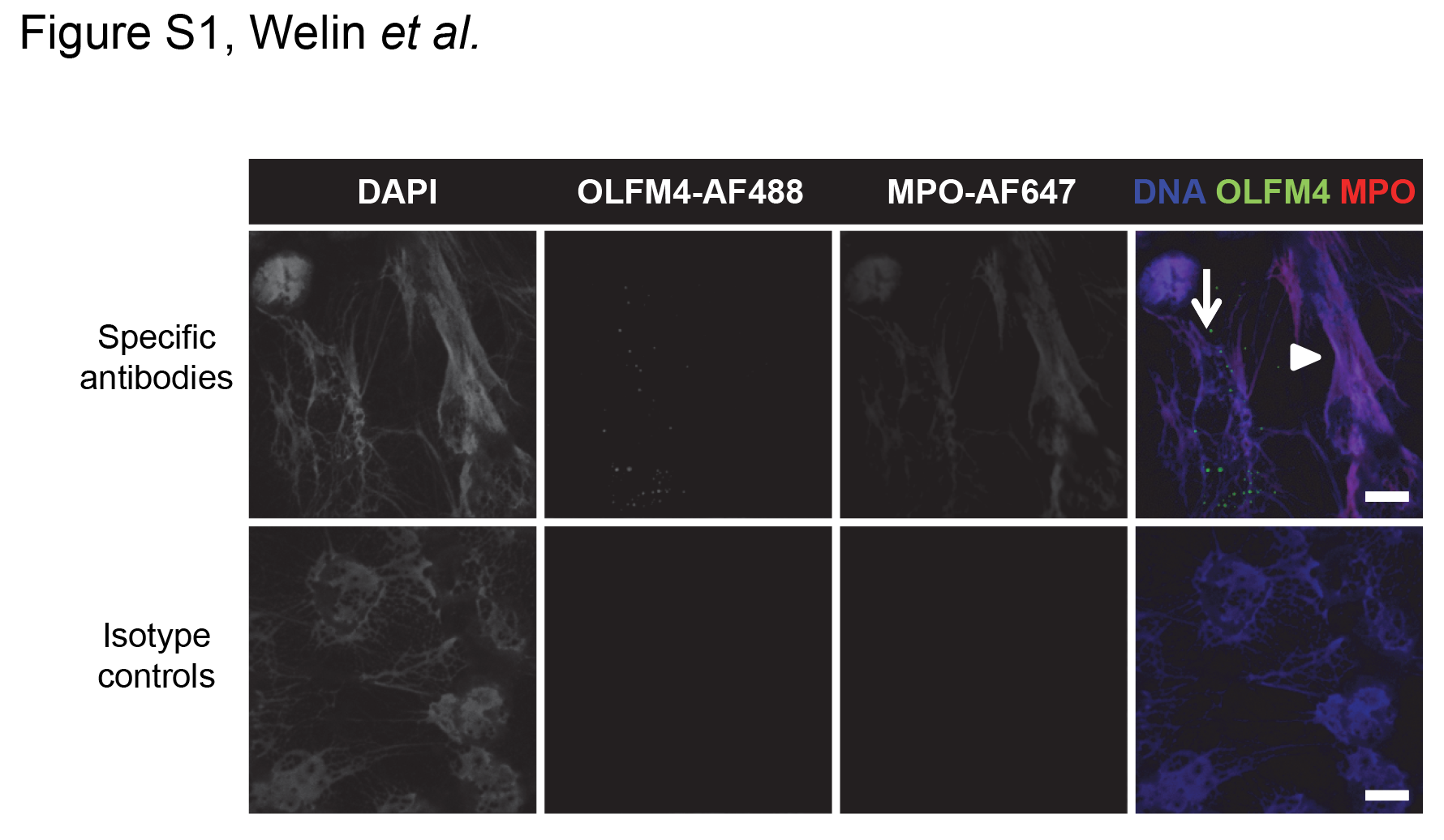

Supplement: Figure S1 — Neutrophils isolated from heparinized blood were adhered to glass coverslips and stimulated with PMA to form NETs. Unpermeabilized samples were immunostained for OLFM4 (green) and MPO (red) (top panel). Alternatively, isotype controls followed by secondary antibody was used (bottom panel). DNA was stained with DAPI (blue). The arrow indicates OLFM4-containing NETs, while the arrow head indicates NETs without OLFM4. The fluorophore conjugates used for each staining are indicated (AF = Alexa Fluor). The scale bars represent 5 µm. (TIF) [file pone.0069575.s001.tif]
